# Implementing the 4R and 9H regimens for TB preventive treatment in Indonesia

**DOI:** 10.5588/ijtld.21.0318

**Published:** 2022-02-01

**Authors:** L. Apriani, R. C. Koesoemadinata, M. L. Bastos, D. A. Wulandari, P. Santoso, B. Alisjahbana, M. E. Rutherford, P. C. Hill, A. Benedetti, D. Menzies, R. Ruslami

**Affiliations:** 1 TB Working Group, Infectious Disease Research Center, Universitas Padjadjaran, Bandung, Indonesia; 2 Department of Public Health, Faculty of Medicine, Universitas Padjadjaran, Bandung, Indonesia; 3 Outcomes Research and Evaluation, Research Institute of the McGill University Health Centre, Montreal, QC, Canada; 4 Department of Child Health, Universitas Padjadjaran/Dr Hasan Sadikin General Hospital, Bandung, Indonesia; 5 Department of Internal Medicine, Faculty of Medicine, Universitas Padjadjaran/Dr Hasan Sadikin General Hospital, Bandung, Indonesia; 6 Centre for International Health, Department of Preventive and Social Medicine, University of Otago, Otago, New Zealand; 7 Departments of Epidemiology, Biostatistics and Occupational Health, and Medicine, McGill University, Montreal, QC, Canada; 8 Department of Biomedical Sciences, Faculty of Medicine, Universitas Padjadjaran, Bandung, Indonesia

**Keywords:** TB preventive treatment, rifampicin, isoniazid

## Abstract

**BACK GROUND:**

The implementation of tuberculosis preventive treatment (TPT) is challenging especially in resource-limited settings. As part of a Phase 3 trial on TPT, we described our experience with the use of rifampicin for 4 months (4R) and isoniazid for 9 months (9H) in Indonesia.

**METHODS:**

In 2011–2017, children and adults with latent TB infection were randomised to either 4R or 9H and followed until 16 months after randomisation for children and 28 months for adults. The primary outcome was the treatment completion rate. Secondary outcomes were Grade 3–5 adverse events (AEs), active TB occurrence, and health costs.

**RESULTS:**

A total of 157 children and 860 adults were enrolled. The 4R treatment completion rate was significantly higher than that of 9H (78.7% vs. 65.5%), for a rate difference of 13.2% (95% CI 7.1–19.2). No Grade 3–5 AEs were reported in children; in adults, it was lower in 4R (0.4%) compared to 9H (2.8%). The incidence of active TB was lower with 4R than with 9H (0.09/100 person-year vs. 0.36/100 person-year) (rate difference: −0.36/100 person-year). The total cost per patient was lower for the 4R regimen than for the 9H regimen (USD151.9 vs. USD179.4 in adults and USD152.9 vs. USD206.5 in children)

**CONCLUSIONS:**

Completion and efficacy rates for 4R were better than for 9H. Compared to 9H, 4R was cheaper in all age groups, safer in adults and equally safe in children. The Indonesian TB program could benefit from these benefits of the 4R regimen.

## Abstract (fr)

**CONTEXTE:**

La mise en place du traitement préventif contre la TB (TPT) est difficile, notamment dans les pays à ressources limitées. Dans le cadre d’un essai de Phase 3 évaluant le TPT, nous avons décrit notre expérience d’utilisation de la rifampicine pendant 4 mois (4R) et de l’isoniazide pendant 9 mois (9H) en Indonésie.

**MÉTHODES:**

En 2011–2017, des enfants et adultes atteints d’infection tuberculeuse latente ont été randomisés soit dans le groupe 4R soit dans le groupe 9H. Ils ont été suivis pendant 16 mois après randomisation pour les enfants et 28 mois pour les adultes. Le critère de jugement principal était le taux d’achèvement du traitement. Les critères de jugement secondaires étaient les évènements indésirables (AE) de grade 3–5, le développement d’une TB active et les coûts de santé.

**RÉSULTATS:**

Au total, 157 enfants et 860 adultes ont été sélectionnés. Le taux d’achèvement du traitement 4R était significativement plus élevé que celui du traitement 9H (78,7% vs. 65,5%), pour une différence des taux de 13,2% (IC 95% 7,1–19,2). Aucun AE de grade 3–5 n’a été rapporté chez l’enfant. Chez l’adulte, ce taux était plus faible sous 4R (0,4%) que sous 9H (2,8%). L’incidence de la TB active était plus faible sous 4R que sous 9H (0,09/100 personnes-années vs. 0,36/100 personnes-années) (différence des taux : −0,36/100 personnes-années). Le coût total par patient était plus faible pour le schéma 4R que pour le schéma 9H (151,9 $ US vs. 179,4 $ US chez l’adulte et 152,9 $ US vs. 206,5 $ US chez l’enfant).

**CONCLUSIONS:**

Les taux d’achèvement du traitement et d’efficacité pour le schéma 4R étaient meilleurs que ceux du schéma 9H. Par rapport au schéma 9H, le schéma 4R était moins onéreux dans toutes les tranches d’âge, plus sûr chez l’adulte et aussi sûr que le schéma 9H chez l’enfant. Le programme indonésien de lutte contre la TB pourrait tirer profit des avantages du schéma 4R.

Indonesia has the second highest incidence of TB in the world, at a rate of 312 per 100,000 population in a country of 271 million people.^1^ It is estimated that around 120 million people (46%) in Indonesia have latent TB infection (LTBI), which means that Indonesia has the third highest LTBI burden in the world.^2^ TB preventive treatment (TPT) is one of the three main preventive strategies recommended by the WHO.^1^ Previous Indonesian national guidelines recommended TPT with 6 months isoniazid (INH, H) only in children aged <5 years who are household contacts of bacteriologically confirmed TB cases, and persons living with HIV.^3^ In 2019, only 9.4% and 12% respectively of these two groups received TPT in Indonesia.^1^

Updated WHO guidelines recommend that TPT be considered for all household contacts of bacteriologically confirmed pulmonary TB cases in high TB incidence countries.^4^ These guidelines recommend the following options for TPT regardless of HIV status: 6 or 9 months of daily INH, a 3-month regimen of once weekly rifapentine (RPT) plus INH, a 3-month regimen of daily INH plus rifampicin (RIF, R). A 1-month regimen of daily rifapentine plus INH or 4 months of daily RIF alone are other possible alternatives.^4^ Shorter and safer regimens are preferred.^1^ Based on these recommendations, Indonesia recently updated the TPT guidelines to treat household contacts and other high-risk groups of all ages.^5^ Information regarding the acceptability and tolerability of 4R regimens (as one of the shorter regimens) in high-risk groups of all ages in Indonesia could therefore benefit the Indonesian National TB Programme in the implementation of TPT. As part of a large randomised trial,^6^,^7^ we describe here our experience in using 4R and compared its completion rates with 9 months of INH (9H) in high-risk groups of all ages in Indonesia. Grade 3–5 adverse events (AEs), occurrence of active TB and health system costs for the two regimens were also evaluated.

## METHODS

### Study design and population

This was part of a multi-centre, open-label, Phase 3, randomised controlled trial.^6^,^7^ Children (0–17 years) and adults (≥18 years) were randomly assigned to receive either 4R or 9H. The trial was conducted in four high-income countries (Australia, Canada, Saudi Arabia and South Korea), two middle-income countries (Brazil and Indonesia) and three West African countries (Benin, Ghana and Guinea). The trial was registered at ClinicalTrials.gov; for adults: NCT00931736 and for children: NCT00170209. The protocol of the main trial is available at https://www.mcgill.ca/tb/projects.

### Study participant and setting

Eligible persons included household contacts of confirmed active pulmonary TB patients treated in lung clinic or one of 30 community health centres (CHC), healthcare workers employed in the CHC who had direct contact with TB patients, people living with HIV or those living with diabetes mellitus (DM) treated in Hasan Sadikin Hospital, a tertiary referral hospital in Bandung, Indonesia. We classified the subjects into two categories: 1) close contacts, defined as those having contact with the index case for ≥4 h/week; and 2) casual contacts, those who had contact with a TB case for <4 h/week.

Potential participants were referred to the research TB clinic from January 2011 to December 2014, and screened for LTBI using a structured questionnaire, physical examination, tuberculin skin test (TST) or a interferon-gamma release assay (IGRA) and chest X-ray. TST- or IGRA-positive participants with normal chest X-ray were eligible for randomisation. Potential participants were excluded if they had a past history of active TB, were exposed to an active TB patient whose isolates were resistant to either trial drug, were currently or planned to become pregnant, receiving any medications with potential serious drug interaction with either trial drug or had a history of allergy to either trial drug. Potential participants with signs and/or symptoms of active TB underwent further examination (physical examination and sputum microscopy) and were referred back to their original healthcare provider to receive full TB treatment if they had active TB. Children aged <5 years who had a household contact with active TB were eligible as long as they did not have active TB, regardless of their TST results.

### Randomisation

Randomisation was computer-generated centrally (McGill University, Montreal, QC, Canada) using a web-based system with an assignment ratio of 1:1 to either 4R or 9H in blocks of varying length (2–8 subjects). Participants within the same household were allocated to the same trial arm if they were all enrolled within the same week.

### Study procedures

After randomisation, all participants were asked to visit the TB research clinic monthly for the first 2 months, and every 2 months (minimum) thereafter. Blood count and liver transaminase levels were checked before treatment in both adults and children and for adults only at the first follow-up visit, unless otherwise indicated. At each follow-up visit, participants were asked to bring the remaining doses for pill counts. Participants were interviewed and examined for signs of AE at each visit. Suspected AE were investigated, treated and reported according to a standardised protocol.

Children aged <5 years with negative TST results (induration size: 0–4 mm) prior to randomisation had repeated TST 8 weeks after the end of household exposure to active TB. The trial drug was continued if they were found to be TST-positive, and discontinued if still TST-negative.

Post-treatment follow-up began after the treatment was completed or discontinued until 16 months after randomisation in children and 28 months in adults. This was done by phone, visits at the TB research clinic or during home visits every 3 months. Participants were asked about TB symptoms and screened for active TB, as indicated. The final diagnoses of Grade 3–5 AE and active TB were made by independent review panels who were blinded to randomisation.

### Ethical considerations

Written informed consent was obtained prior to enrolment. This trial was approved by the Biomedical Clinical Research Ethics Board of the McGill University Health Centre Research Institute, Montreal, QC, Canada; and the Universitas Padjadjaran Ethics Committees, Bandung, Indonesia (No.23/FKUP-RSHS/KPEK/Kep./EC /2010).

### Sample size consideration

The main trial included 6,063 adult participants to assess non-inferiority in TB prevention effectiveness, and 844 children to assess the safety and completion rates in the two study arms.^6^,^7^ For adults, the total of 3,283 participants in each study group provided >80% power to detect non-inferiority for TB prevention and >95% power to detect differences in AE and completion rates. For children, 411 participants per group provided 80% power to conclude that the 4R regimen was not inferior to the 9H regimen in terms of AEs. The trial site in Bandung, Indonesia, aimed to recruit a total of 1,000 children and adults. The total of 356 participants per group provided 80% power and α = 0.05 to detect a 10% better completion rate in 4R, with 60% of participants having completed 9H.

### Study outcomes

Primary outcome was treatment completion rates. Secondary outcomes included study acceptance rates, indications, Grade 3–5 AEs, active TB and health system costs. Study acceptance rates were estimated from the number of participants who underwent randomisation as numerator and the number of eligible participants as denominator. Indications for TPT were categorised as follows: close or occasional exposure to confirmed active TB, HIV infection and other immunosuppressive condition or therapy (DM, etc.). Grade 3–5 AEs that led to permanent discontinuation of the trial drug was defined as per protocol. Confirmed active TB was defined as a positive culture for *Mycobacterium tuberculosis* or a finding of caseating granulomas in a biopsy specimen obtained from any organ. As reported previously,^8^ we included all costs related to initial screening/medical evaluation, study drugs, routine follow-up visits (including assessments of adherence and potential AE), as well as evaluation and management of possible AE or active TB under health costs.

### Data management and statistical analysis

Descriptive statistics were performed using Stata^®^ v14.2 (Stata Corp LP, College Station, TX, USA). Proportions and 95% confidence intervals (CIs) of study acceptance rates, indications and completion rates were estimated using a generalised linear mixed model with binary outcome accounting for clustering by household. The cumulative incidence of Grade 3–5 AEs during treatment were calculated in both study arms. Rates of active TB per 100 person-years were calculated in both study arms. Risk or rate differences were estimated between the study arms overall and for adults and children separately, with 95% CIs accounting for clustering by household. For health cost analysis, the frequency of each health care activity for each study participant was multiplied by the unit cost of that activity. The costs for each activity were then summed to provide a total cost per participant, which was used to calculate the total and mean cost for participants who met different study endpoints. We calculated a ratio of mean costs per participant randomly assigned to 4R divided by the mean cost per participant assigned to 9H to provide a summary estimate of the relative costs for the two regimens. The cost comparison analysis was performed separately for adults and children included in the modified intention-to-treat (MITT) analyses. In Indonesia, the TPT regimen was 6 months of INH (6H); to take this into account, we performed a sensitivity analysis comparing health cost for 4R vs. 6H. For this analysis, we censored all health services used more than 213 days after randomisation (180 days plus 18% of additional time allowed for completion per protocol) in the group receiving 9H.

## RESULTS

**Figure 1:**
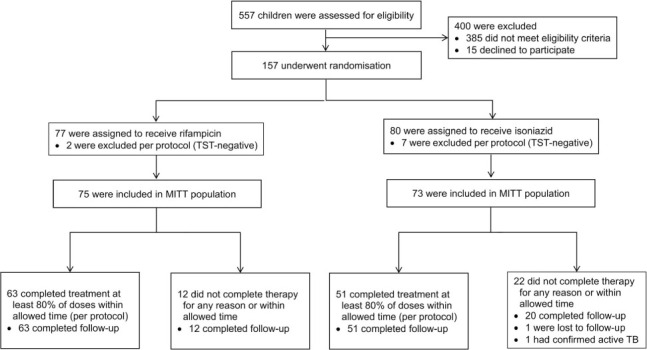
Enrolment and treatment completion in child contacts. TST = tuberculin skin test; MITT = modified intention-to-treat.

**Figure 2:**
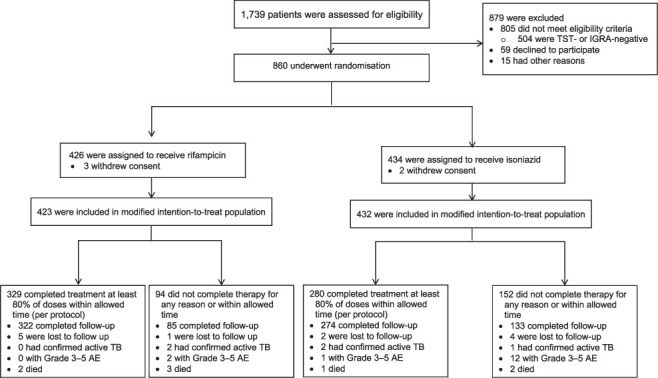
Enrolment and treatment completion in adults. TST = tuberculin skin test; IGRA = interferon-gamma release assay; AE = adverse event.

We screened 557 children and 1,739 adults as potential participants. Of the 172 children and 934 adults who met the inclusion criteria, 15 children (8.7%) and 59 (6.3%) adults declined to participate (Figures 1 and 2), yielding study acceptance rates of 91.3% in children and 92.1% in adults. Nine children were excluded after randomisation because they were TST-negative result on repeat testing at 8 weeks, so that 148 children remained in the MITT analysis and 146 children were followed up until 16 months after randomisation. Of the 860 adults who underwent randomisation, five withdrew consent, so that 855 adults remained in the MITT analysis and 814 adults were followed up until 28 months after randomisation.

Participant characteristics are shown in Table 1. The median age was 40 years, and 34.1% were males. Of all the participants, 39 (3.9%) were aged <5 years. More than 70% of the participants were close contacts of confirmed active TB cases, and 282 (28.1%) had at least one other family member who participated in this trial. Twenty-one were HIV infected adults and one quarter (*n* = 245) were healthcare workers. The treatment completion rate for 4R was significantly higher than that for 9H (difference: 13.2%, 95% CI 7.1–19.2; Table 2). The most common reason for not completing the trial therapy was the participants’ decision to discontinue the trial drug early without evidence of any AE.

**Table 1:** Characteristic of the participants at baseline: the modified intention-to-treat population

Characteristic	4R (*n* = 498) *n* (%)	9H (*n* = 505) *n* (%)	Total (*n* = 1,003) *n* (%)
Age, years, median [IQR]	40 [25–50]	38 [25–48]	40 [25–49]
Age group, years			
Children (≤17 years)	75 (15.1)	73 (14.5)	148 (14.8)
0–4	19 (3.8)	20 (4.0)	39 (3.9)
5–12	37 (7.4)	26 (5.1)	63 (6.3)
13–17	19 (3.8)	27 (5.4)	46 (4.6)
Adults (≥18 years)	423 (84.9)	432 (85.5)	855 (85.2)
≥18–35	126 (25.3)	151 (29.9)	277 (27.6)
36–50	182 (36.6)	180 (35.6)	362 (36.1)
51–90	115 (23.1)	101 (20.0)	216 (21.5)
Male sex	170 (34.1)	172 (34.1)	342 (34.1)
Height, m, median [IQR]			
Children (≤17 years)	1.3 [0.9–1.5]	1.4 [1.0–1.5]	1.3 [1.0–1.5]
Adults (≥18 years)	1.5 [1.5–1.6]	1.5 [1.5–1.6]	1.5 [1.5–1.6]
Weight, kg, median [IQR]			
Children (≤17 years)	27.4 [15.2–41.4] 30.1	[15.8–41.6]	28.6 [15.8–41.5]
Adults (≥18 years)	58.0 [50.0–66.0] 58.0	[50.0–64.5]	58.0 [50.0–65.0]
Body mass index, kg/m^2^, median [IQR]			
Children (≤17 years)	16.2 [15.2–19.5] 17.0	[15.3–18.4]	16.9 [15.3–18.9]
Adults (≥18 years)	24.2 [20.7–27.8] 24.0	[21.0–27.5]	24.0 [20.8–27.7]
Reaction size on tuberculin skin test, mm			
<5 (child case contacts)	8 (1.6)	5 (1.0)	13 (1.3)
5–9	10 (2.0)	13 (2.6)	23 (2.3)
10–14	171 (34.3)	184 (36.4)	355 (35.4)
≥15	309 (62.1)	303 (60.0)	612 (61.0)
Risk factors as indication for treatment			
Confirmed active TB in close contact	343 (68.9)	372 (73.6)	715 (71.3)
Confirmed active TB in casual contact	131 (26.3)	114 (22.6)	245 (24.4)
HIV infection	11 (2.2)	10 (2.0)	21 (2.1)
Other immunosuppressive condition^*^	13 (2.6)	9 (1.8)	22 (2.2)
Result on chest radiography			
Normal	345 (69.3)	358 (70.9)	703 (70.1)
Abnormality not related to TB	129 (25.9)	131 (25.9)	260 (25.9)
Hilar lymph node enlargement	12 (2.4)	6 (1.2)	18 (1.8)
Other possible TB-related stable abnormality	12 (2.4)	10 (2.0)	22 (2.2)
Family member in trial	140 (28.1)	142 (28.1)	282 (28.1)

^*^ Included diabetes mellitus, tumour necrosis factor-alpha inhibitor therapy, renal failure.

IQR = interquartile range.

**Table 2:** Completion of treatment in adult and child participants in the modified intention-to-treat population

Variable	4R (*n* = 498) *n* (%)	9H (*n* = 505) *n* (%)	Difference % (95% CI)	*P* value
Treatment completed: ≥80% of doses (total)	392 (78.7)	331 (65.5)	13.2 (7.1 to 19.2)	<0.001
Children (≤17 years)	63 (84.0)	51 (69.9)	14.1 (−0.05 to 28.3)	0.05
Adults (≥18 years)	329 (77.8)	280 (64.8)	13.0 (6.7 to 19.2)	<0.001
Treatment not completed for any reason	106 (21.3)	174 (34.5)	−13.2 (−19.2 to −7.1)	<0.001
Never started treatment	3 (0.6)	5 (1.0)	−0.4 (−1.3 to 0.6)	0.42
Therapy stopped permanently for adverse event	7 (1.4)	15 (3.0)	−1.6 (−3.3 to 0.2)	0.088
Diagnosis of active TB during treatment period	1 (0.2)	0 (0.0)	—	—
Started but patient decided to stop treatment early^*^	95 (19.1)	154 (30.5)	−10.2 (−15.9 to −4.6)	<0.001
Took 50–79% of doses	13 (2.6)	17 (3.4)	−0.7 (−2.9 to 1.4)	0.49
Took 1–49% of doses	82 (16.5)	137 (27.1)	−10.7 (−16.2 to −5.1)	<0.001

* These condition were drop-out, non-adherence and lost to follow-up.

4R = 4 months of rifampicin; 9H = 9 months of isoniazid; CI = confidence interval.

No Grade 3–5 AEs were reported in children; in adults, Grade 3–5 AEs were significantly less frequent with the 4R regimen than with 9H (difference: −2.5%, 95% CI −4.3 to −0.8; Table 3).

**Table 3:** Treatment adverse events and occurrence of active TB among children and adult participants: the modified intention-to-treat population

Description	4R (*n* = 498) *n* (%)	9H (*n* = 505) *n* (%)	Difference % (95% CI)	*P* value
Grade 3–5 adverse event, total^*^	2 (0.4)	13 (2.8)	−2.2 (−3.6 to −0.7)	0.004
Children (≤17 years)	0 (0.0)	0 (0.0)	—	
Adults (≥18 years)	2 (0.5)	13 (3.0)	−2.5 (−4.3 to −0.8)	0.004
Active TB				
Total number of person-years of follow-up	1,075	1,093		
Children (≤17 years)	0	1	—	
Adults (≥18 years)	1	3	—	
Number of active TB cases/100 person-year (95% CI)	0.09	0.36	−0.36 (−0.72 to −0.007)	0.03

* Adverse event resulted in the permanent discontinuation of the trial drug.

4R = 4 months of rifampicin; 9H = 9 months of isoniazid; CI = confidence interval.

Active TB was diagnosed in one participant of the 4R arm (rate: 0.09/100 person-years, equivalent to 0.21 over the 28-month period) and four in the 9H arm (rate: 0.36/100 person-years, equivalent to 0.83 over the 28-month period). The total number of participants developed active TB was significantly lower in 4R than in 9H (difference: −0.36/100 person-year, 95% CI −0.72 to −0.007, equivalent to −1.66 to −0.02 over the 28-month period). The only child who developed active pulmonary TB, was HIV-negative and discontinued after receiving less than 10% of the assigned dose of 9H because of the participant’s decision. Of the four adults who developed active TB, two completed, and two others did not complete study treatment. Two of the adults with active TB were HIV-infected persons on antiretroviral therapy. One adult had pulmonary TB, while the other three developed extrapulmonary TB (two with TB lymphadenitis and one with TB spondylitis).

The total health costs, including drugs, personnel and laboratory testing, per MITT participant among adults and children were significantly lower in the 4R group than in the 9H group (Table 4). The mean (4R to 9H) ratio of the total cost per patient was 0.85 (95% CI 0.81–0.88) in adults and 0.74 (95% CI 0.65–0.80) in children. The most important contributors to costs were routine follow-up visits, LTBI drugs, blood tests and AEs. In sensitivity analysis (Table 5), we compared costs for 4R to those for 6H, the TPT regimen recommended by the Indonesia National TB Programme. Despite higher cost of the drug component of 4R, total health costs were lower with 4R due to reduced visits, blood tests and AE care. Costs were non-significantly lower with 4R than 6H among adults, and significantly lower with 4R in children.

**Table 4:** Costs (in USD): MITT population in adult and children population

	Adults (≥18 years)			Children (<17 years)		
	
	4R costs Mean ± SD	9H costs Mean ± SD	Ratio of mean costs 4R/9H (95% CI)	4R costs Mean ± SD	9H costs Mean ± SD	Ratio of mean costs 4R/9H (95% CI)
Pre-randomisation evaluation	65.5 ± 1.7	65.4 ± 0.37	1.0	68.8 ± 0.10	68.8 ± 0	1.0
Follow-up during treatment						
Participants in MITT analysis, *n*	423	432		75	73	
Participants who completed treatment, *n*	309	269		63	51	
Drugs (INH or RIF only)	19.2 ± 8.2	3.8 ± 2.1	5.05	15.1 ± 7.2	15.6 ± 16.6	1.0
Follow-up visits	66.3 ± 23.7	105.9 ± 49.4	0.62	64.8 ± 14.9	117.9 ± 45.9	0.54
Follow-up tests and procedures	9.8 ± 5.3	10.4 ± 5.9	0.9	4.2 ± 1.1	4.1 ± 0.8	1.0
Costs for AE care	0.9 ± 11.5	4.2 ± 47.6	0.21	—	—	—
Total costs						
Total costs/MITT patients	151.9 ± 30.8	179.4 ± 66.9 0.85	(0.81 to 0.88)	152.9 ± 18.2	206.5 ± 59.5	0.74 (0.65 to 0.80)
Total costs/patients who completed treatment	164.6 ± 12.9	207.6 ± 21.7 0.79	(0.78 to 0.81)	157.2 ± 13.3	234.7 ± 48.6	0.67 (0.63 to 0.72)

USD =United States dollar; MITT =modified intention-to-treat; 4R=4 months of rifampicin; SD =standard deviation; 9H= 9 months of isoniazid; CI =confidence interval; INH = isoniazid; RIF = rifampicin; AE = adverse event.

**Table 5:** Sensitivity analysis comparing cost (in USD) for 4R vs. 9H: MITT population in adult and children population

	Adults (≥18 years)			Children (<17 years)		
	
	4R costs Mean ± SD	9H costs Mean ± SD	Ratio of mean costs 4R/9H (95% CI)	4R costs Mean ± SD	9H costs Mean ± SD	Ratio of mean costs 4R/9H (95% CI)
Baseline evaluation	65.5 ± 1.7	65.4 ± 0.37	1.0	68.8 ± 0.10	68.8 ± 0	1.0
Follow up evaluation						
Participants in MITT analysis, *n*	423	432		75	73	
Drugs (INH or RIF only)	19.2 ± 8.2	2.7 ± 1.5	7.1	15.1 ± 7.2	10.6 ± 11.17	1.4
Follow-up visits	66.3 ± 23.7	74.3 ± 24.6	0.89	64.8 ± 14.9	85.0 ± 25.8	0.76
Follow-up tests and procedures	9.8 ± 5.3	10.3 ± 5.9	0.95	4.2 ± 1.1	4.10 ± 0.86	1.0
Costs for AE care	0.9 ± 11.5	4.1 ± 47.6	0.22	—	—	—
Total costs						
All patients/events	151.9 ± 30.8	156.8 ± 53.4	0.97 (0.93 to 1.01)	152.9 ± 18.2	168.54 ± 35.0	0.91 (0.84 to 0.96)

USD =United States dollar; 4R =4 months of rifampicin; 9H=9 months of isoniazid; MITT=modified intention-to-treat; SD =standard deviation; CI =confidence interval; INH = isoniazid; RIF = rifampicin; AE = adverse event.

## DISCUSSION

In a clinical trial in urban Indonesia, we showed that the acceptance rate in participating in a trial and receiving TPT among high-risk TB groups was >90%. The 4R regimen had a higher treatment completion rate, fewer Grade 3–5 AEs and better efficacy compared to 9H group, and lower total costs per person compared to 9H. In addition, the difference in total health system costs between 4R and 6H was significant in children, but was not significant in adults.

In this study, acceptance rates for participating and receiving TPT were high in both arms. However, although TPT is covered by the National TB Programme, it remains a low priority in busy health facilities in Indonesia. This is due to several challenges which are commonly found worldwide, such as knowledge gaps in healthcare providers and concerns regarding side effects of TPT.^9^ In this trial, study physicians and nurses were given ample time to explain the benefit and risks of TPT to the participant during the socialisation meeting at the CHC and prior to interviewing each participant at the clinic. We provided printed information material to read, and gave participants opportunities to ask questions and discuss with their families; we also provided a phone number in case further advice was needed. By improving communication, we were able to substantially increase the acceptance of TPT.

The completion rate was higher in the 4R group than in the 9H group. This finding is consistent with other studies, which reported a significantly higher rate of treatment completion in the shorter RIF-based regimens (3R or 4R) than with 6H or 9H.^10^–^16^ In our study, the most common reason for not completing treatment was participants’ decision to discontinue the trial drug before an adequate number of doses had been taken, and this was mostly because participants were busy at work and had no time for follow-up visits and taking medication.

Among adult participants, Grade 3–5 AEs that resulted in the discontinuation of the trial drug were more frequent with 9H than 4R. This finding is in line with other study findings: RIF-based regimens (3R or 4R) are associated with lower rates of hepatotoxic effects among adults.^12^,^13^,^15^

We also noted that the occurrence of active TB was lower in the 4R group than in the 9H group. Other evidence suggests equivalent effectiveness, but not superiority, of shorter regimens.^13^,^17^ Our findings add new evidence that 4R is more effective than 9H. Compared to 9H (6H), TPT with 4R resulted in lower costs for children and adults in Indonesia. Our result is similar to evidence from other settings.^18^

This is the first randomised study of its kind in Indonesia. High acceptance and completion rates may reflect the greater time dedicated to patient education and motivation by the research staff, which may be difficult to achieve in routine care. The limitations of this study included the open-label design, which may led to bias in the ascertainment of treatment completion or AE. However, all the final diagnoses of Grade 3–5 AEs and active TB were made by blinded independent review panels. Furthermore, using the pill count to determine treatment completion is not a highly reliable method.^19^,^20^ We may have overestimated costs due to the more intensive followup in a study environment. Also, costs were not estimated from the patient’s perspective. However, patient costs are likely to be lower in the 4R arm as the fewer number of visits and tests will have resulted in lower indirect (time) and direct (out-of-pocket) costs per patient.

When implementing the 4R regimen in Indonesia, not only drug procurement costs but also overall health system costs should be considered. The higher costs of pills should not prevent the adoption of 4R by Indonesia or other resource-limited settings. Study results provides evidence to suggest that if Indonesia updates the TPT guidelines to treat household contacts and other high-risk groups of all ages, the 4R regimen could have several benefits in the Indonesian setting—superior completion rates, efficacy and safety, with lower healthcare costs.
